# Perceptions of young people living with HIV and caregivers in Botswana on analytical treatment interruption studies: a qualitative approach

**DOI:** 10.1080/25787489.2026.2673739

**Published:** 2026-05-20

**Authors:** Catherine K. Koofhethile, Poloko Kebaabetswe, Ontlametse T. Choga, Fikile Ditshweu, Pearl Ntshonga, Bonolo R. Molefe, Oganne Batlang, Patrick Mokgethi, Tsholofelo Mosielele, Phenyo Sabone, Pearl Kgoreletso, Amantle Gouwe, Keitumetse Mosepele, Rashida Majola, Dorcas Maruapula, Kaelo Seatla, Wonderful Choga, Gbolahan Ajibola, Joseph Makhema, Gaerolwe Masheto, Xu Yu, Sikhulile Moyo, Mathias Lichterfeld, Shahin Lockman, Roger Shapiro

**Affiliations:** a Botswana Harvard Health Partnership, Gaborone, Botswana;; b Department of Immunology and Infectious Diseases, Harvard T.H. Chan School of Public Health, Boston, MA, USA;; c Ragon Institute of MGH, MIT and Harvard, Cambridge, MA, USA;; d Division of Postgraduate Research, University of Venda, Thohoyandou, South Africa;; e Infectious Disease Division, Brigham and Women's Hospital, Boston, MA, USA

**Keywords:** ATI, perceptions, young-people living with HIV, qualitative, HIV cure research

## Abstract

**Background:**

Current research suggests that post-treatment HIV control may be possible for some people following antiretroviral treatment (ART) interruption. Although closely monitored analytic treatment interruption (ATI) studies can be performed safely and are crucial to determine who may no longer require ART, currently it is unknown how acceptable these studies will be in Botswana.

**Aim:**

To determine the knowledge, attitudes and acceptability of ATI-inclusive studies in Botswana.

**Methods:**

We employed a non-randomized purposive design to recruit study participants. We conducted face-to-face open-ended interviews with 12 young people living with HIV and 8 caregivers, 8 healthcare providers and 4 community advocates in Gaborone and Palapye.

**Results:**

Thirty-two (32) participants consented to be interviewed. The median age of young people living with HIV was 29 years. All young people living with HIV indicated that they were on ART and acknowledged the benefits of being on ART. All young people living with HIV were unaware of ATIs, whereas 6 out of 8 caregivers, 6 out of 8 healthcare providers and 2 out of 4 community advocates had never heard of ATIs. Most participants found ATI research to be acceptable and believed that it would be beneficial provided there were adequate risk mitigations in place.

**Conclusions:**

ATI studies were acceptable to both young people living with HIV and their caregivers. There was limited knowledge about ATI, highlighting a need to inform communities and health care providers on the scientific aspects of ATI and HIV cure research.

## Background

While antiretroviral therapy (ART) has proven to successfully improve the life expectancy of people living with HIV, the search for a cure is ongoing. The current obstacle to curing HIV is its persistence in the body as a reservoir of latently infected CD4+ T cells. These reservoir cells can be activated following cessation of ART, leading to viral rebound [1,2]. However, recent research suggests that post-treatment control is possible for some people living with HIV following analytical treatment interruption (ATI) [3,4]. An ATI study is defined as temporary cessation of ART under thorough clinical and laboratory care [5]. The nature of these studies guide HIV cure research, seek to identify ART-free viral control mechanisms, and explore how effective new treatment strategies and interventions are [6]. They also help in characterizing how immune mechanism may be involved in achieving and maintaining viral suppression post-intervention [7].

Since the rollout of ART in Botswana in the early 2000s, public health messaging has emphasized lifelong ART without interruption. This messaging is based on the expected 70+ year persistence of the latent reservoir in adults [8] and increased risk of drug resistance if ART is non-continuous [9,10]. ATI studies are crucial to perform to identify individuals who may no longer require ART, and are now considered to be safe with close monitoring for viral rebound. Selected people living with HIV who may be able to stop ART in the setting of an ATI study include: (1) those who may have been misdiagnosed in early life, when false positive testing may be more frequent [11]; (2) those who initially tested positive but no longer show evidence for intact (replication-competent) HIV in their reservoirs; and (3) those who have very low viral reservoirs or locked viral reservoirs (HIV integrated mostly in non-encoding regions of the genome) and can control any small amount of HIV replication with their own immune system [12,13]. Before enrolment in ATI studies, it is essential to understand the different perceptions of people living with HIV, caregivers of people living with HIV and stakeholders in ATI-inclusive studies. Previous work has reported ATI perceptions in high-burden regions and indicated that young people living with HIV expressed concerns regarding risks during blood draws, mental health issues and potential coercion to participate in ATI studies [14]. However, in the same study, young people living with HIV highlighted that engaging in clinical research will give them better access to care and HIV education [14].

How acceptable ATI studies will be in Botswana is currently unknown. It is also not known how to prevent misperceptions, including therapeutic misconceptions, whereby participants may interpret experimental remission outcomes as indications of a definitive HIV cure [15]. These misperceptions may lead to individuals to stop ART on their own outside of the research context. Furthermore, while data from the region suggest that young populations can tolerate the psychological uncertainty of ATI, success is heavily dependent on addressing anxieties regarding viral rebound and potential transmission to partners [16]. For caregivers, the decision-making process is further complicated by the burden of increased clinical monitoring and fears of treatment failure [17].

Understanding the perceptions and attitudes regarding ATI among young people living with HIV and caregivers of people living with HIV can help identify gaps that need to be addressed prior to the implementation of ATI studies. Therefore, we conducted an in-depth qualitative open-ended interview study with young people living with HIV, caregivers of people living with HIV, healthcare providers and community advocates to understand these gaps in the Botswana context.

The main aim of this study was to determine participants' knowledge, understanding, attitudes, and perceptions of ATI studies. The study addressed the following specific objectives:

- To determine young people living with HIV's knowledge of HIV, as well as their understanding of the benefits and challenges of ART.
- To describe participants' knowledge of ATI studies.
- To describe the participants' attitudes and perceptions of ATI as a potential first step towards stopping ART in participants with the potential for functional HIV cure.
- To describe participants' perceptions of the potential benefits, risks, and challenges of discontinuing ART during ATI studies.
- To describe the participants' fears/concerns about the discontinuation of ART during ATI studies, for themselves, their patients, or the community.
- To determine the feasibility and acceptability of ATI implementation among people living with HIV.

## Methodology

### Study design

This study employed a non-randomized purposive method involving qualitative design to enable the researchers to describe the views and perceptions of young people living with HIV and caregivers of people living with HIV regarding ATI-inclusive studies in Botswana. The study was led by behavioural scientists with extensive research experience in qualitative studies. The data were collected by two research assistants who were trained in the study protocol, ethics and interview skills. Key interview domains in the interview guide (Supplementary Material 1) included areas shown in Table 1.

### Study population

We focused on four groups of people representing both peri-urban and urban populations in Botswana: (1) young people living with HIV aged 18 years and above attending Infectious Disease Care Clinics (IDCC) in Gaborone and Palapye. (2) Caregivers of people living with HIV—which includes parents or caregivers of children living with HIV and taking treatment at IDCC study sites; (3) health care providers, including doctors and nurses directly involved with the care of young people living with HIV at the respective sites; and (4) community advocates, such as community leaders and community advisory board (CAB) members, as well as high-level officers from the Ministry of Health, representing policy makers. These participants were recruited and interviewed between June and August 2024.

### Study sites

The study was conducted in the Southern (Gaborone) and Central region (Palapye), where we selected one IDCC in a peri-urban area (Palapye) and one clinic representing an urban area (Gaborone). Each IDCC selected had adequate monthly attendance of at least 100 people living with HIV.

### Ethical considerations

The study was conducted in accordance with the Code of Federal Regulations on the Protection of Human Subjects (45 CFR Part 46) and institutional research policies and procedures. IRB approval was received from the Botswana Ministry of Health—Human Research Development Committee (HRDC). All personnel involved in the conduct of this study completed the Human Subjects Protection Training. Study participants provided oral informed consent.

### Recruitment

We approached 36 potential participants and recorded the response rate for those enrolled. A systematic non-randomized approach was used to recruit young people living with HIV while waiting for their services or at the end of their check-up visits. Research assistants recruited young people living with HIV who were 18 years and above. Young people living with HIV were first informed about the aim of the study, and if they were interested in taking part, they were taken to a private room, and the informed consent process started, followed by an interview. Sampling young people living with HIV from two diverse clinics from different geographical sites on different days provided us with a diverse study sample with varied experiences and perceptions.

Health care providers were recruited from the IDCC clinics, and parents of children with HIV were recruited with the help of the IDCC staff, who provided the data collectors with a list of potential participants. Participants were informed of the aim of the study and, if interested, an appointment was set for informed consent and interviewing.

Recruitment of the community advocates was conducted through a ‘snowball’ methodology where the researcher identified one potential community advocate who also identified a potential participant in the community who worked with or worked in an organization that supports young people living with HIV. Like the other participants who were recruited, the potential participants were called and informed about the study and its aim. If they were interested, they were asked to either visit the IDCC clinic or to be interviewed at their preferred place and time. All the recruited community advocates agreed to take part in the study.

All participants provided verbal consent and permission to audiotape before the interview commenced. In addition to verbal consent, young people living with HIV signed a disclaimer form, which emphasized that their decision to participate in the study did not mean that they should stop taking their ART. The informed consent conversation took place in either Setswana or English according to the participants' preferences. Study participants were provided with BWP100 (~$7) for their transport reimbursement.

### Data collection

Data collection used a semi-structured open-ended interview guide developed by the research team based on the study objectives. We employed a face-to-face in-depth individual interview. The interviews were conducted by local research assistants trained in the protocol, interview guides, and ethics. Interviews were conducted in a private location, audio recorded and spanned 30–45 min. Interviews were transcribed verbatim, and those in Setswana were translated and back translated to facilitate data analysis. Key interview domains in the interview guide included; (1) demographic information, (2) knowledge of young people living with HIV via ART, (3) knowledge of ATI, (4) perceptions of ATI, (5) potential risks and benefits of ATI, (6) fears and concerns about ATIs, and (7) ATI acceptance.

### Data analysis

The data analysis team employed the technique of code mapping using ATLAS.ti 7. The process allowed the research team to generate transcript marking segments corresponding to different codes, which were summarized into the different codes, themes, and quotations of different participants.

## Results

### Characteristics of study participants

We enrolled 32 (89%) of the 36 individuals approached (four young people living with HIV, but no adults, declined participation). Among those included, there were 12 young people living with HIV (six Gaborone, six Palapye), eight caregivers of children living with HIV (four Gaborone, four Palapye), eight health care providers/Ministry of Health HIV managers (four Palapye, four Gaborone), and four community advocates (two Palapye, two Gaborone) (Table 2). Young people living with HIV had a median age of 29 (interquartile range [IQR]: 26–31) years while caregivers, healthcare providers and community advocates had a median age of 43 (IQR: 37–49) years. Most participants were female (67% of people living with HIV and 70% of caregivers). The median duration on ART for young people living with HIV was 11 (IQR: 5–19) years (Table 2).

The participants were grouped into youth living with HIV, caregivers, healthcare providers, and community advocates with the following corresponding codes:

**PY01–PY06:** Palapye Youth living with HIV.
**GY01–GY06:** Gaborone Youth living with HIV.
**PC01–PC04:** Palapye Caregivers.
**GC01–GC04:** Gaborone Caregivers.
**PH01–PH04:** Palapye Health Care Providers.
**GH01–GH04:** Gaborone Health Care Providers.
**PCA01–PCA02:** Palapye Community Advocates.
**GCA01–GCA02:** Gaborone Community Advocates.

### Knowledge of young people living with HIV on ART

All young people living with HIV indicated that they were on ART and provided their duration on ART, and acknowledged the benefits of being on ART, such as stability, improving health, lowering of viral load, and achieving and maintaining viral suppression which reduces the risk of HIV transmission to a partner.

Most young people living with HIV from Palapye mentioned several challenges that came with being on ART which included: lengthy times at health facilities; negative effects on life in general and experiencing stigma and discrimination. With respect to the issue of discrimination, one participant stated, *‘the moment people see you walk through the hospital corridor leading to the ARV dispensary, already they start looking at you in some kind of way and judging you’* (PY01) ‘Even at school, we are *discriminated by other students*’ (GY03).

In Gaborone, half of the young people living with HIV said they had experienced challenges, including defaulting on treatment, depression, fear of the unknown, and chronic conditions, such as kidney failure and high blood pressure. Young people living with HIV in both Palapye and Gaborone mentioned the following as ways of dealing with their challenges: self-counselling, counselling from health care providers, social and parental support, self-determination to take pills as advised, self-acceptance that they are living with HIV and are on ART, setting an alarm to remind them to take treatment, rescheduling the time to take ART, and believing in God.

### Knowledge of ATI

All participants were asked about their knowledge of ATIs. This question was preceded by an introduction of ATI as an interventional research program where HIV treatment is interrupted among people living with HIV under close monitoring after taking treatment for a long time with no evidence of replication of the virus or in cases where their own immune system is able to control virus replication. All young people living with HIV indicated that they had never heard of ATI. All the participants mentioned that they never thought it was possible to interrupt ARVs. The statements included, *‘I did not have any information, I did not think it was possible, I thought it was a taboo to stop taking treatment’* (PY01). Similarly, the majority of caregivers in both Gaborone and Palapye had never heard of an ATI; a few had heard about ATI at a workshop on injectable ART. Those who had heard about ATI thought it was possible to put some people living with HIV off ART for complications related to ART but had never heard that ART could be stopped in people living with HIV to test for post-treatment control. When asked about their knowledge of ATI, half of the community advocates in Palapye had not heard about ATI; however, the other half were aware of treatment interruption but not the analytical part. PCA01 said ‘*treatment interruption is a case where a person who is on ART stops taking it for a specific reason but as for analytic, I do not know’*. However, they expressed that ATI could bring hope for a cure, as stated by a community advocate: ‘*When you listen to the news you hear that it is possible for some people to be suspended from the medication and be monitored and this brings hope for treatment to be stopped one day’* (GCA02).

### Perceptions of ATI

When asked if ART should be life-long, young people living with HIV in Palapye and Gaborone differed. Half of the young people living with HIV in Palapye said it should be lifelong. The support for lifelong ART was based on socioeconomic differences, as some youths might not have enough food to boost their immunity, and the fact that some might engage in risky behaviours that could increase the risk of HIV infection. This was highlighted by a participant in Palapye, who said, ‘*It is not all of us who are able to find nutrients that would help boost our immune system, and some might think that because they have stopped taking ARVs, they can now start sleeping around’* (PY04). Those who did not support the need for life-long ART indicated that there might be a goal for ending the treatment: *‘I just believe that when you are taking some pills, there should be a time to stop them and you continue with a different treatment, or you totally stop because they have done their work in the body*’ (GY05). In support of this opinion, PY02 said *‘the main reason why we take ARVs is for the immune system to be strong to be able to fight the virus, the body needs nutrients and if you don't eat well the virus will thrive and so I strongly believe that if a person eats healthy and exercise, there is no need for them to take the pills for life their bodies are able to fight against the virus’*.

When asked if ART should be life-long, the majority of caregivers felt that ART is not a cure. As for the caregivers in Gaborone, half agreed that ART should not be lifelong, especially when the CD4 count was high. Health care providers' perceptions of whether ART should be life-long in Palapye differed, while half believed that ART should be lifelong, as mentioned by PH03: *‘The treatment at the moment is not necessarily a cure, we have not yet reached that point; we have been following the research done on ARVs, so I have not come across anything that speaks about the cure or complete eradication of the virus in the body’* (PH03). In support of PH04, *‘We wish and hope that a cure be found. If there is no cure then lifelong’*.

All the community advocates in Palapye mentioned that ART should be lifelong, but some answers were conditional, for example, PCA01 said, *‘yes it should be life-long unless there are new developments that can state otherwise,’* while others were clear that it should be life long as they had been taught so. PCA02 said, ‘*We have been taught that the only way to live long is when you are taking treatment that would suppress the virus so if you are not taking any treatment your viral load goes up and your CD4 count low, and you will get sick and eventually die’*. As for the community advocates in Gaborone, they had mixed feelings on whether ART should be life-long or not, for example, GCA02 suggested that ART should be life-long. She stated, ‘*they can be lifelong, the reason I am saying this is that as people we suffer from other illnesses like diabetes and Bp and if going through all that I don't think it will be a good idea to stop and be monitored as already you have other complications which can risk your health but when it is just HIV without other diseases yes then treatment can be stopped and one is monitored’*.

Although the recurring theme was that ART should be lifelong, some participants felt that this should only apply to those with risky behaviours; one caregiver stated that *‘I feel it has to be a life time thing in those people who have careless behaviours that are putting themselves at high risk of being sick, others who are adhering to their treatment and behave well maybe to those, ART should not be lifelong’* (GH04). Some did not support the need for life-long ART, stating, ‘*It should not be lifelong, I go for checkups only because it is a requirement, but everything is in place, the CD4 count is ok, so I am here wondering why I am still taking ARVs when everything is ok*’ (GC01).

### Potential risks and benefits

#### Benefits of ATI

The majority of young people living with HIV believed that ATIs would have benefits including: less stigmatization and thus more employment, a reduction in long queues at health facilities, time savings, cost savings in transportation to health facilities, and the ability to help free socialization with peers. One young person living with HIV said ‘*youth fail to take medication thinking their peers would laugh at them and be seen at the hospitals to collect pills is not something they want to do’*. One youth LWH who did not believe that ATIs would have benefits said ‘*I strongly believe the virus will multiply so I do not believe ATI would bring benefits but rather increase the risk of infection even when there is monitoring, it would put them at risk and if things do not go according to the plan the virus will multiply’* (PY01).

Similarly, the majority of community advocates believed that ATI would have benefits, as highlighted by a caregiver who said *‘the biggest benefit is hope, it is the hope that the process is creating and one of the biggest burdens we saw in 80s and 90s was hopelessness in the society and more people died because of fear of being infected than the actual physical infection’* (GCA01). In support, a participant in Palapye PCA02 agreed that ATI would reduce pill burden and said ‘*Again putting the medicine in your body daily truly speaking is like you are putting poison in your body and will keep doing it until you die’*. Just like youths, most caregivers in Palapye and Gaborone agreed that ATI would benefit young people on ART and that ATI would help the youth to live like others, without worrying about taking ARVs daily.

Many health care providers in Palapye believed that ATI would be beneficial to the youths and would reduce the burden of monitoring and workload, and parents would not miss community meetings. They mentioned that ATI would have a psychological impact on patients on ART and that they would no longer take lots of pills and pill fatigue would reduce. To emphasize this point, GH01 said, ‘*If I have 1000 patients and half of them are off treatment that means the workload will be reduced'* In support GH02 added by saying ‘*I think benefits include the reduction of cost and adverse events’*.

#### Potential risks of ATI

Regarding the risks of ATI, young people living with HIV mentioned weakness in the body when ART is paused: ‘*If you may take two days without taking them you would feel that your body is not reacting well now I don't know about going years without taking them’* (PY05), and the risk of HIV infection and other opportunistic infections: ‘*The doctors said if you stop taking ARV, there is a high change for you to contract other diseases like TB, cancer and would die’* (PY02). Similarly, most youths in Gaborone agreed with the risks mentioned by youths in Palapye; however, a few disagreed that they could be risks of ATI, as they would be closely monitored.

The majority of caregivers highlighted similar risks: ‘*Yes, I think there could be risks, people are going to have some fears, we were told that ARVs are for life and should never be stopped and have seen people who defaulted becoming sick*’ (PC02). They also indicated that if ARVs were stopped, there would be more resources to treat other infections. Some caregivers in Palapye who indicated that they would be no risk cited confidence in the doctors that they would not advise them to stop ART when they know it would not be safe, as was echoed by

PC04, *‘I don't believe there could be any risk because the doctors know what they will do to prevent them”*. This was supported by GC01, who said that *‘When they follow all the guidelines it won't be risky’*. However, some care givers said that ATI would bring confusion because some young people on ART but not on ATI would be tempted to stop since their friends have stopped as indicated by a caregiver who said ‘*Some would hear about the possibility of stopping ARV's and decide to stop on their own before going to the health officials to get the full information and the risk to this is that most people would default when their immune system is weak, and it becomes something else’* (GCO3).

As for the health care providers, the minority agreed with participants, who said that they would be no risks, as highlighted by PH01, who said *‘I do not foresee any problem it is only because we have not yet reached there, fear of unknown’;* however, the majority indicated that they would be risks in the implementation of ATI. Examples of risks mentioned included increased opportunistic infections and an increased number of babies born with HIV, leading to greater costs, as more money would be spent on medication to treat these infections. Interestingly, all the health care providers in Gaborone agreed that there are risks associated with introducing ATI and mentioned the same risks mentioned by the health care providers in Palapye but added that a change in messaging that ART could be stopped would cause confusion and mistrust among them.

The community advocates both in Gaborone and Palapye agreed with the youths, the caregivers, and the health providers that there would be risks associated with introducing ATI. In addition to the risks mentioned by other participants, the community advocates talked of the difficulty in finding the right language to communicate what ATI is all about without causing confusion; one of the participants (GCA01) stated that ‘*first risk is that of finding the language that can adequately communicate this without causing confusion second, and related to this is that of what you know are wanting to say being misunderstood for what you are not saying that for me is the major risk’*.

### Fears and concerns about ATIs

The majority of young people living with HIV highlighted their fears about ATI as fear of the unknown, potential increased opportunistic infections, body changes concerns, such as weight loss, fear of increased mental problems due to stopping ART, increased infections due to poor immunity, misconceptions that ATI is a cure, and that youth may engage in risky behaviours, such as stopping ART on their own. Similarly, caregivers raised concerns about the development of HIV drug resistance, as the public would think the government has run out of funds and thus reduce the support to people living with HIV and conflicting messaging about the need for lifelong ART. A third of the participants who said they did not have fears and concerns referenced their confidence in the nurses and doctors who would not give them something that would not be good for their health.

Health care providers in Palapye indicated that ATI would lead to increased opportunistic infections and mortality rate would increase, fears of passing infections, such as TB to caregivers, and confusion because, while other patients would be on ARV, others would not. In Gaborone, the common theme was fear of resistance and that the public would think the government has run out of funds and thus reducing the support to patients.

The community advocates expressed differing fears. The majority of both Gaborone and Palapye were concerned about possible increases in infections and misconceptions about ATI as a cure and that since youths are engaged in risky behaviours, when their friends who are also on ART learn that it is possible to stop them, they may be tempted to stop ARV on their own. To address these concerns, advocates recommended that:

- ATI should be performed only on adults who have been on treatment for a long time and not on children less than 18 years, as their behaviours are unpredictable.
- ATI should be accompanied by counselling and education to facilitate acceptance, and counselling should be done based on individual needs and packaged in a way that caters to urban and rural settlements.

### ATI acceptance

Overall, the majority of young people living with HIV accepted ATI and would be willing to participate in ATI studies. Common reasons for acceptance included relief from pill burden and side effects of ARVs, reduced hospital visits, and confidence that doctors and nurses would assess them before telling them to stop treatment. One youth who would reject ATI expressed her dilemma: ‘*I am conflicted, taking pills is tiring, part of me wants to but looking at what we have been taught, a part of me says no’* (PY01). Fear of the side effects of ARVs was also echoed as a reason to accept ATI, as was expressed by participant GY05, who said, ‘*I will participate but will need counselling because I am afraid that without them will get worse, but I am convinced that ARV contributed to by kidney failure, but besides this, I am willing to try ATI, as taking a lot of pills is painful’*.

Half of the caregivers in Gaborone indicated that their children would be willing to participate in ATI studies due to pill fatigue. The remaining half, who indicated that their children would not participate due to fear of increased viral load in the absence of ARV and the fact that their children had been taught that ARVs are for life and that it would not be easy to convince them otherwise, as cited by participant GC03 who said, ‘*my child will not agree to participate, he has been taught at Baylor that ARVs are for life’*.

Generally, half of the caregivers in both Palapye and Gaborone believe that their children or the people there are caring for would be willing to take part in ATI while the other half did not believe that their children or relative there is care would be willing to take in ATI.

When asked if they would be in favour of their children or relatives to participate in ATI, most caregivers in Palapye indicated that they would not be in favour of their children to participate because of fear that their children's immune system would become weak and that parents would not know what would happen when ARVs were stopped. This was emphasized by PC03, who said, *‘Even if there is monitoring, I would still not agree as I don't know what would happen after treatment is stopped as the body would be used to taking pills daily’*. On the contrary, all care givers in Gaborone would be in favour of their children participation in ATI. The common rational was that ATI would relieve them of the burden of giving their children medications and help them live a better life. Generally, all caregivers in Gaborone would be in favour of their children's participation in AT while in Palapye, the majority would not agree.

It is worth noting that community advocates differed, in Palapye, all did not agree to introducing ATI to children while in Gaborone all agreed to introducing ATI to children and felt that it would benefit them. It is not known why such a difference.

## Discussion

To our knowledge, this is the first study to evaluate the perceptions and acceptability of performing ATI studies in Botswana in the context of HIV cure research. We identified a high level of acceptance of ATI by young people living with HIV, caregivers, health care providers and community advocates, with specific hesitations expressed across all groups. Knowledge regarding ATI was limited, demonstrating a need to inform communities and providers on the scientific aspects of ATI and HIV cure research, particularly on the clinical and psychosocial risks of ATIs.

The overall acceptance of ATI in the study among young people living with HIV, caregivers, health care providers, and community advocates was higher than that reported in some prior studies from Africa. Hesitation regarding ATI has been noted in Ghana and South Africa. Among participants in a study conducted in Ghana [18], 44% of 251 survey participants indicated that they would be willing to take part in ATI and HIV cure studies. Similarly, a study conducted in South Africa interviewed 15 stakeholders and concluded that there was a low level of awareness of HIV cure research; hence, this may influence the low rate of participation in ATIs [19]. However, in a study conducted in Ghana, there was a willingness to participate in HIV cure research through blood drawing and being involved in clinical trials [18]. Our data are more in accordance with research findings from the United States and recent findings from the FRESH cohort in South Africa, where people living with HIV were willing to stop ART to participate in HIV cure research with close physician monitoring [19,20]. The reasons for the lack of ATI acceptability appear to be consistent across studies, even when the magnitude of acceptability differs and includes limited knowledge about ATIs, the proven improvement in quality of life provided by ART, and concerns regarding transmission to sexual partners and in the community [19,21]. Worrying about ATI and its link with HIV transmission has been observed frequently in prior studies [22], including those among HIV sero-different couples [23–25]. This universal concern highlights the need to understand HIV transmission dynamics among people living with HIV who are enrolled in ATI studies for HIV cure research. Despite extensive evaluation of perception about ATI in developed countries, it appears that each setting may also be unique, highlighting the importance of local and regional research and engagement involving people living with HIV, caretakers, and community members. An ongoing challenge is to provide information in layman's language to ensure that communities fully understand the complex concepts and implications involved in ATI research [17,21]. On balance, the studies to date highlight that there may be mixed messages regarding how ATI studies are perceived in different contexts.

In our study, the acceptance of ATI was mostly influenced by a few key themes. First, ART has always been promoted as lifelong because of the persistence of HIV in latent reservoir in adults [8]; the consistent use of ART has been associated with good health outcomes and limited drug resistance [9,10], and all individuals were aware of poor outcomes when defaulting from ART. Second, the enrolled individuals were concerned that an ATI goes against the ‘U = U’ (undetectable = untransmissible) concept and may increase the risk of HIV transmission. Third, a lack of knowledge about ATI was of great concern. It has been shown in previous studies that people would be willing to join ATI-inclusive studies provided that there is adequate information communicated in nontechnical terms [26]. None of the young people living with HIV had heard of an ATI, while some indicated that they never imagined that stopping ART could be possible in their lifetime. While caregivers in the Palapye area indicated that they had never heard of ATI, half of those in Gaborone city had heard of ATI. Finally, the participants acknowledged other benefits from ATI, which included reducing stigma, reducing the financial burden resulting from lifelong ART, and perceived reduction of time needed at health facilities.

## Study limitations

Although the study was conducted at two locations and among several groups of people, the sample size utilized may not have completely reflected the true perception of ATIs in Botswana. This could be resolved with a larger sample size. Another limitation was the inadequate knowledge regarding ATI prior to the information provided by our research team, which could have affected the findings. A possible risk was that people living with HIV might feel that they could stop ART on their own, but we addressed this by ensuring that all young people living with HIV who participated in the study were on ART and understood the benefits of being on treatment and that this study did not imply that they could stop treatment on their own or as part of the study.

## Conclusion

Our findings provide insight into how people living with HIV in Botswana view ATIs, which is essential to inform HIV cure studies involving ATIs that are planned in the country. Despite the limited knowledge across the groups we studied, young people living with HIV in both urban and peri-urban areas, and caregivers throughout the community, health care providers and community advocates, had a favourable impression of ATI studies provided that there was adequate clinical monitoring and that other risk mitigations were in place. This limited knowledge highlights the urgent need to engage communities and providers in the scientific aspects of ATIs and HIV cure research.

## Supplementary Material

Supplementary

## Figures and Tables

**Table 1.** Key interview domains.

Key interview domains	Interviewees			
	Young-PLWH	Caregivers	Health care providers	Community advocates
ART understanding	X			
Knowledge on ATI	X	X	X	X
Perceptions	X	X	X	X
Benefits, risks, challenges	X	X	X	X
Fears and concerns	X	X	X	X
ATI acceptance	X	X	X	X
Demographic information	X	X	X	X

**Table 2.** Participant demographic and social characteristics.

Group	Variable	Category	*N* = 32	%	Median (IQR)
Young PLWH			12		
	Age				29 (26–31)
	Sex				
		Male	4	33.3	
		Female	8	66.6	
	Years on ART				11 (5–19)
	Education level				
		Form 2	1	8.3	
		Form 3	3	25	
		BGCSE	5	41.6	
		Tertiary	3	25	
	Residence				
		Gaborone	6		
		Palapye	6		
Caregivers			8		
	Age				42.5 (39–45.5)
	Sex				
		Male	0	0	
		Female	8	100	
	Relation to PLWH				
		Parent	6	75	
		*Other* (relative, spouse)	2	25	
	Employment status				
		Employed	2	25	
		Unemployed	4	50	
		Self-employed	2	25	
	Residence				
		Gaborone	4	50	
		Palapye	4	50	
Healthcare providers			8		
	Age				45 (38.5–45.75)
	Sex				
		Male	4	50	
		Female	4	50	
	Institution				
		Palapye IDCC	4	50	
		Princess Marina IDCC	3	37.5	
		Ministry of Health	1	12.5	
	Profession				
		Nurses	5	62.5	
		Medical doctors	3	37.5	
	Location				
		Palapye	4	50	
		Gaborone	4	50	
Community advocates			4		
	Age				44.5 (35.75–55.25)
	Sex				
		Male	1	25	
		Female	3	75	
	Institution				
		Non-governmental organization	3	75	
		Religious leader	1	25	
	Location				
		Palapye	2	50	
		Gaborone	2	50	
