# Supplementary material for: Perceptions of young people living with HIV and caregivers in Botswana on analytical treatment interruption studies: a qualitative approach
Source: HIV Res Clin Pract. Author manuscript; Available in PMC 2026 Aug 5. (PMC13439527; doi:10.1080/25787489.2026.2673739)
Supplement: Supplementary file 1 [file NIHMS2194071-supplement-Supplementary.pdf]

Title: Perceptions of young adults living with HIV (LWH), caregivers/advocates, and health care providers on the planned Analytical Treatment Interruption (ATI) study in Botswana- A qualitative approach

INTERVIEW GUIDE

YOUTH (PLWH) -ONLY

PTID:

Date: Location:

The following questions should serve as a guide for interviews with young people living with HIV(PLWH). Before the interviews begin, the consent form should be reviewed and signed.

Introduction

Thank you so much for agreeing to participate in this interview today. My name is _________, and I am working with a team that conducts a highly controlled Analytical Treatment Interruption (ATI) study to determine the perceptions of different groups of people on the planned ATI initiative in Botswana. I would like to ask you a few questions about your perceptions on ATI. The interview will take approximately 30-45 minutes. Please feel free to answer all the questions asked.

Before we begin, I will read you a short background of our study so that you understand what the kind of question I am going to ask you:

Since ART rollout in Botswana in the early 2000s, the messaging is that it always needs to be lifelong, and that it should never be stopped. This messaging was based on previous research conducted that suggested that HIV is able to stick around the body for many years even when a person living with the virus is on suppressive treatment and the concern for development of antiretroviral resistance. However, as the science around treatment interruption and the ART regimens used have advanced, the messaging surrounding lifelong ART – especially in the context of carefully controlled clinical trials – is now changing. Especially in children , there are several potential groups who may be able to stop ART if this is performed under carefully controlled circumstances within an ATI study. These groups include: 1) those who may have been misdiagnosed in early life, when false positive testing may be more frequent; 2) those who initially tested positive, but no longer show evidence for intact (replication competent/potentially infectious virus) hidden in different parts of the body (in the reservoirs); and 3) those who have very low viral reservoirs, or locked viral reservoirs (HIV only in non-encoding regions of the genome), and can control any small amount of HIV replication by their own immune system.

Based on this background, I am now going to start the interview, is it ok?

1. **ARV Understanding**

1.1 Are you taking ART if Yes which ones?

1.2 How long have you been taking ART?

1.3 What are the benefits of taking ART?

1.4 Is there anything you would like to tell me about challenges you have face taking ART if any?

1.5 What has been helpful in dealing with challenges of taking ART all the time?

2. **Knowledge on ATI**

2.1 Before my introduction on ATI, what was your awareness and understanding of the Analytical Treatment Interruption (ATI)?

2.2 What have you heard about ATI? Tell me what you know about it?

3. **Perceptions**

3.1 According to you, should ART be life long? why or why not?

3.2 There are several potential groups who may be able to stop ART if this is performed under carefully controlled circumstances. Do you think children living with HIV should be in these groups? if yes why, if not why?.

3.3 As a young person living with HIV and have taking been on ART for a long time what will be your initial reactions to the idea that children who have been taking ART for a long time could be allowed to stop them while there are under close monitoring of their viral load?

3.4 What role do you think ATI may have as part of the ART management in the IDCC clinics in Botswana?

4. **Benefits, risks, and challenges**

4.1 According to you, are they any benefits of ATI? if so, please explain in detail the benefits to you as a child living with HIV, (probe adherence, stigma) be, if there no benefits explain why?

4.2 Can ATI benefit caregivers, and the community? If so, how? If cannot benefit them why not?

4.3 Are they potential risks to introducing ATI to you and other children living with HIV? If so, what are the risks ? (Probe: rebound risk,)

4.4 What challenges do you anticipate when ATI is implemented to children living with HIV and have been taking treatment for a long time (probe: challenges from peers, public)

4.5 What recommendations/advise could you give to address the challenges you have talked about?

5. Fears and concerns

5.1 As a young person living with HIV and having been on ART for a long time, do you have any fears / concerns about the discontinuation of ART during the ATI?

6. ATI Acceptance

6.1 If you were selected to participate in ATI, would you be willing to participate, why or why not?

7- Demographic information

7.1 Age:

7.2 Gender:

7..3 Education level:

7..4 Number of years on ART:

We have come to the end of the interview.

Thank you very much for your time.
